# Levodopa-Induced Dyskinesias in Parkinson’s Disease: An Overview on Pathophysiology, Clinical Manifestations, Therapy Management Strategies and Future Directions

**DOI:** 10.3390/jcm12134427

**Published:** 2023-06-30

**Authors:** Lazzaro di Biase, Pasquale Maria Pecoraro, Simona Paola Carbone, Maria Letizia Caminiti, Vincenzo Di Lazzaro

**Affiliations:** 1 Fondazione Policlinico Universitario Campus Bio-Medico, Via Alvaro del Portillo, 200, 00128 Roma, Italy; p.pecoraro@unicampus.it (P.M.P.); s.carbone@unicampus.it (S.P.C.); mlcaminiti@gmail.com (M.L.C.); v.dilazzaro@unicampus.it (V.D.L.); 2 Brain Innovations Lab, Università Campus Bio-Medico di Roma, Via Álvaro del Portillo, 21, 00128 Rome, Italy; 3 Unit of Neurology, Neurophysiology, Neurobiology and Psichiatry, Department of Medicine and Surgery, Università Campus Bio-Medico di Roma, Via Alvaro del Portillo, 21, 00128 Roma, Italy

**Keywords:** Parkinson’s disease, motor complications, levodopa-induced dyskinesias, LIDs, pathophysiology, clinical manifestations, therapy management

## Abstract

Since its first introduction, levodopa has become the cornerstone for the treatment of Parkinson’s disease and remains the leading therapeutic choice for motor control therapy so far. Unfortunately, the subsequent appearance of abnormal involuntary movements, known as dyskinesias, is a frequent drawback. Despite the deep knowledge of this complication, in terms of clinical phenomenology and the temporal relationship during a levodopa regimen, less is clear about the pathophysiological mechanisms underpinning it. As the disease progresses, specific oscillatory activities of both motor cortical and basal ganglia neurons and variation in levodopa metabolism, in terms of the dopamine receptor stimulation pattern and turnover rate, underlie dyskinesia onset. This review aims to provide a global overview on levodopa-induced dyskinesias, focusing on pathophysiology, clinical manifestations, therapy management strategies and future directions.

## 1. Introduction

After the description of the pathological correlate of parkinsonian syndrome, characterized by the degeneration of the dopaminergic neurons of the substantia nigra pars compacta (SNc) [1], the main goal of Parkinson’s disease (PD) treatment is to restore dopaminergic transmission [2,3]. Although several options to improve synaptic dopaminergic transmission are available, such as dopamine receptor agonists, COMT inhibitors and MAO-B inhibitors, levodopa still remains the most effective pharmacological treatment for PD [3].

Abnormal involuntary movements, or dyskinesias, along with motor fluctuations, are motor complications of chronic levodopa treatment and neurodegeneration, causing impairment in the quality of life [4]. Even in the first descriptions of levodopa effects in PD patients, in 1967-69, Cotzias and Papavasiliou [5] described the presence of involuntary movements after levodopa administration, during the peak of therapeutic effect [2] in particular in patients with the longest disease duration. The pathophysiology of dyskinesias is still far from being clearly elucidated. The key risk factors for developing such abnormal involuntary movements are levodopa dose and disease duration/severity [6,7]. The non-physiologic intermittent pulsatile stimulation of dopamine (DA) receptors and the subsequent changes in proteins and genes produce alterations in the neuronal firing patterns [8,9,10], which are well-known dyskinesia-contributing factors. 

The application of invasive neurophysiological techniques opened the way to closely recording neuronal firing activity [11]. Specific oscillatory activities of both cortical areas and basal ganglia neurons superimposed to dyskinesia appearance have been described, reinforcing the hypothesis of a pivotal role of such oscillations in the genesis of levodopa-induced dyskinesias (LIDs) [12,13,14,15,16,17,18,19]. Recognizing troublesome dyskinesias is crucial for the movement disorder specialist, since their presence is considered a marker of advanced Parkinson’s disease and an indicator of the eligibility for device-aided therapy [20]. 

In the present review, we aimed to summarize the current state of the art in the field of LIDs. A global overview on LIDs is provided, with a focus on the most recent evidence about clinical aspects, pathophysiology and neurophysiology. Conventional strategies for the therapeutic management of LIDs along with new possible therapeutic approaches are reported. 

## 2. Levodopa-Induced Dyskinesias 

### 2.1. Clinical Risk Factors 

The main risk factors for developing LIDs are younger age, female sex (biased by a lower weight), longer disease duration, longer PD duration before the initiation of levodopa, more advanced disease and the dose and duration of levodopa treatment. Studies in the literature agree that levodopa therapy carries a higher risk than DA [6,7]. An RCT, ELLDOPA study, demonstrated how patients receiving a higher levodopa dosage (>300 mg) showed a significantly higher risk of developing LIDs [21]. Abnormal involuntary movements do not occur exclusively with levodopa therapy: dopamine agonists [22,23,24,25,26,27], COMT inhibitors [28] and MAO inhibitors [29] could be correlated with such abnormal movements. 

### 2.2. Genetic Risk Factors 

Because LID susceptibility varies greatly from patient to patient, it is possible that genetic factors contribute to the development of LID. Genes involved in dopamine metabolism [30], transport [31] and signaling [32], as well as genes involved in glutamate transmission [33] and synaptic plasticity [34], have been linked to an increased risk of developing LID [35,36].

The following table (Table 1) lists some of the important genes connected to the genetic risk of LID.

A recent systematic review and meta-analysis of genetic factors related to LIDs analyzed 33 studies including a total of 27,092 subjects of different ethnicities. They analyzed 37 genes (22 possibly associated with dyskinesia) and 158 variants (94 possibly related to dyskinesia). The studies reviewed demonstrated inconsistent results, and the meta-analysis failed to demonstrate any association between genetic factors and LID susceptibility [37].

It is crucial to remember that the genetic component of LID is complex and involves the interaction of several genes in addition to the significant influence of non-genetic factors. 

### 2.3. Epidemiology

**Figure 1:**
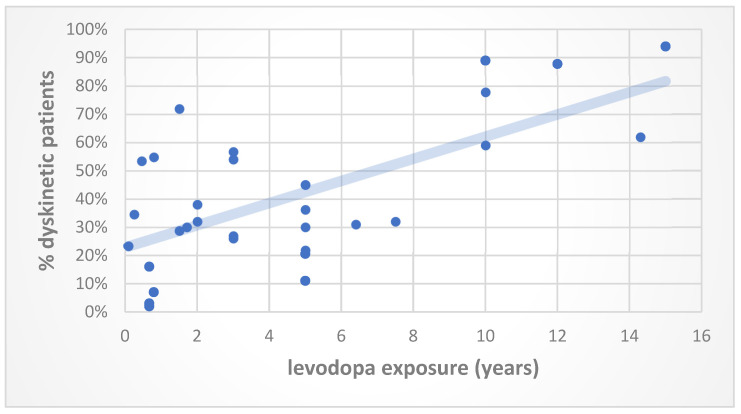
Relationship between the incidence of levodopa-induced dyskinesias and levodopa exposure time. Data in the graph derive from the mean levodopa exposure time from each study reported in Table 2 expressed in years, the blue line represents the trend line for the reported data.

From the first introduction of levodopa, in the late 1960s, abnormal involuntary movements have been reported in about half of patients after 6 months of treatment [38]. Clinical studies [7,39,40], literature meta-analysis [41] and trials [21,22,23,24,28,42,43,44,45] that investigated the phenomenon of levodopa-induced dyskinesias, focusing on the temporal delay between levodopa-based therapy initiation, the starting time of abnormal involuntary movements and the rate of the occurrence of dyskinesias, are expressed as a scatter plot in Figure 1 and listed in Table 2.

In the study of Ahlskog and Muenter [41], the authors analyzed the cumulative frequency of levodopa-induced dyskinesias through a meta-analysis of the literature during discrete intervals of treatment from 1966 to September 2000, including time-series pre- and post-levodopa availability. The authors concluded that patients with a diagnosis of PD prior to levodopa introduction had a higher frequency of levodopa-induced dyskinesias in the early phase of treatment, with 53.4% of them experiencing involuntary movements just 5–6 months after starting levodopa. This contrasts with modern-era series, in which the occurrence of abnormal involuntary movements was negligible for the first 1–2 years on levodopa. At 4–6 years of follow-up, patients that started antiparkinsonian therapy based on a levodopa regimen still had lower median dyskinesia frequencies (36–39%). Conversely, dyskinesias were present in 90% of PD patients in the only three modern-era series that reported the cumulative dyskinesia frequency over a 9-year period of therapy with levodopa. Of note, in a 5-year, double-blind study, PD patients randomized ab initio to levodopa showed a three-fold probability of exhibiting dyskinesias than subjects initially randomized to Ropirinole, and the median time to dyskinesia appearance was significantly longer for the group that firstly assumed the dopamine agonist. After a 5-year follow-up, 45% of patients randomized to initial therapy with levodopa developed involuntary movements, while, after 10 years on levodopa, dyskinesias occurred in 77.8%. Additionally, the median time to dyskinesia onset was 7 years in subjects originally treated with levodopa [24]. It is worth noting that Rajput and Fenton [39] designed the only reported study so far that highlighted the pattern of the motor complications of long-term levodopa treatment in pathologically confirmed PD. The study concluded that after 14.3 years of levodopa exposure, dyskinesias occurred in 61.9% of patients and represented the earliest and most common adverse effects of levodopa. In the ELLDOPA study [21], early PD patients were randomized to receive different daily carbidopa–levodopa regimens up to 150 mg and 600 mg or assigned to the placebo group for a period of 40 weeks, followed by a withdrawal of 2 weeks. The higher the levodopa dose was administered, the higher the incidence of dyskinesias: abnormal involuntary movements occurred in 16% of patients after taking 600 mg/die of levodopa for 8 months compared to 3% of subjects that were on 150 mg/die. Finally, it is worth citing the Sydney Multicenter Study [45] because it was the only study to deeply define the clinical characteristics of dyskinesias, providing information about their severity. In this study, 149 previously untreated PD patients were randomized to initial blinded treatment with bromocriptine or levodopa. Of the 52 patients surviving at least 15 years, 94% experienced dyskinesias. Moreover, 6 of the 52 subjects (12%) had severe involuntary movements with a Unified Parkinson’s Disease Rating Scale (UPDRS) item 33 of 3. Levodopa-induced dyskinesias started after a mean duration of treatment of 4.2 years for the levodopa group. 

### 2.4. Clinical Manifestations

LIDs are arrhythmic, not stereotyped, casual, discontinuous, involuntary movements that can involve any body district and in particular limbs, the head and the trunk. From a phenomenological point of view, LIDs can appear in different ways as choreic, ballistic, athetoid or dystonic movements. They are present at rest and can be increased by voluntary movements, cognitive activities and emotional stressors.

ON-period dyskinesias are more common than OFF-period dyskinesias, are choreiform or choreoathetoid in nature and can sometimes even be ballistic [46]. 

Levodopa-induced dyskinesias can be further classified into peak-dose dyskinesias, occurring during the ON phase in coincidence with the peak plasma level of levodopa and affecting mainly the axial musculature and proximal upper limbs. Square-wave dyskinesias are a particularly severe form of ON dyskinesias: this kind of LID occupies the entire ON phase, and the patient does not experience the ON motor state without dyskinetic movements [47].

Another type of dyskinesia, diphasic dyskinesia [48], emerges at lower levodopa plasma levels during the transitions from ON to OFF phases and from OFF to ON phases, involving lower limbs asymmetrically. Diphasic dyskinesias characteristically affect lower limbs with repetitive rapidly alternating dystonic flexion/extension foot movements or leg kicking in a stereotyped pattern, often associated with high-stepping and bizarre gaits [49]. Additionally, it was found that patients who have never had diphasic dyskinesia run the chance of developing it after starting an intestinal infusion of levodopa/carbidopa monotherapy [50].

**Figure 2:**
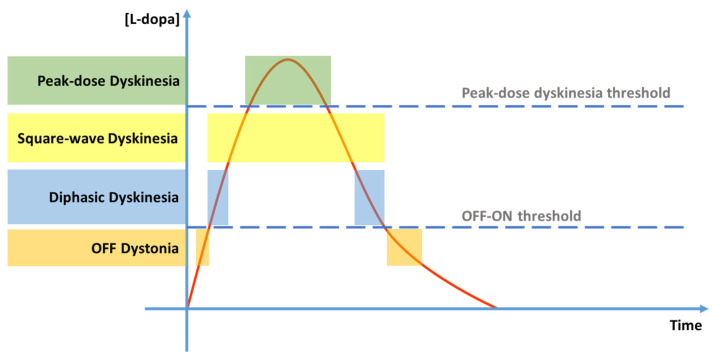
Levodopa-induced dyskinesia types. Legend: red line = plasma levodopa concentration [L-dopa] over time; lower dashed line = levodopa concentration threshold for OFF–ON transition; upper dashed line = levodopa concentration threshold for peak-dose dyskinesia transition; orange boxes = OFF dystonia periods; blue boxes = diphasic dyskinesia periods; yellow box = square-wave dyskinesia period; green box = peak-dose dyskinesia period.

The last mentioned type occurs only during the OFF phase in the phenomenological form of dystonia [46,51,52,53,54] (Figure 2).

### 2.5. Objective LID Monitoring

**Figure 3:**
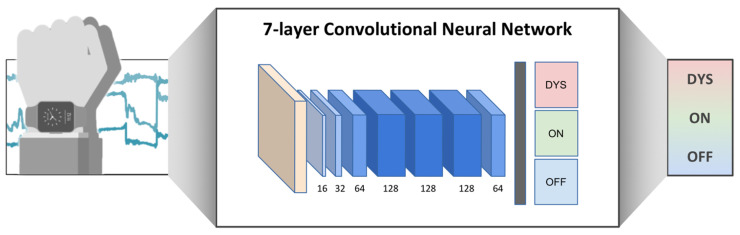
LID and ON/OFF motor status recognition trough convolutional neural network. (Figure drawn by Dr. Pfister, reproduced, under the terms of the Creative Commons Attribution 4.0 License, from [78]).

Clinical evaluation remains the gold standard for motor symptom identification and diagnosis in routine clinical practice. However, new technologies, like wearable motion sensor devices, are opening new ways not only for continuous at-home symptom monitoring [55,56] but also for the objective and quantitative description of PD motor symptoms [57,58,59], like tremors [60,61,62,63], bradykinesia [64,65,66], rigidity [66,67,68,69], gait, balance and postural issues [70,71,72,73,74,75], alongside motor complications like motor fluctuations and dyskinesias [76,77,78]. Additionally, among non-wearable sensors, video-based systems represent a reliable solution to assess the features of LIDs [79]. In addition, data science with the development of artificial intelligence and machine learning algorithms will further improve the diagnostic process [80,81,82], motor symptom identification [83,84,85] and the management and optimization of the therapy to avoid motor complications [86] (Figure 3).

### 2.6. Pathophysiology

#### Levodopa Pharmacokinetics and Pharmacodynamics

Oral levodopa has a bioavailability of 30% because it is highly metabolized into DA by peripheral Aromatic L-amino acid decarboxylase (AADC) expressed in the gut. Concomitant administration of AADC peripheral inhibitors (AADCI) increases the bioavailability of levodopa up to three times, reducing the required therapeutic dose [87]. Levodopa competes with the transport system of neutral amino acids both in the intestinal mucosa and the blood–brain barrier [87]. Meals with a high protein intake increase the plasmatic concentrations of neutral amino acids, reducing the absorption of levodopa and its therapeutic effect [88]; therefore, the levodopa administration regimen should be adapted to the mealtime in order to enhance its therapeutic effect [89]. In addition, mathematical models showed that not all amino acids compete with levodopa absorption and that a serine-rich diet could even improve the bioavailability by 22% compared with the ante cibum administration [90]. The evolution of pharmacokinetic and pharmacodynamic parameters with disease progression has been widely investigated in the literature [87,91,92,93,94,95,96,97]. Variation in pharmacokinetic values in advanced PD stages is controversial: some studies evidenced no change in levodopa kinetics for advancing disease [87,95,96,98,99], while others demonstrated that levodopa pharmacokinetic parameters may be useful to determine disease severity and the duration of Parkinson’s disease [94,100]. For example, in the work of Adamiak and Kaldonska [94], higher Cmax and AUC values were observed in patients with more advanced Hoehn and Yahr stages. In the same study, the authors further demonstrated a correlation between the age of patients and Tmax, while disease duration was directly related to AUC. Moreover, Nyholm [101] hypothesized that a reduced activity of the levodopa metabolizing enzymes may increase the AUC in patients on longstanding levodopa therapy. This is particularly true for the elderly, probably related to a reduced clearance. Conversely, as demonstrated by Contin and Riva [97], the latency of response and the duration of response to a standard levodopa test dose are significantly shortened with disease progression, while the magnitude of the effect is unchanged [98] or even enhanced [87,95,102,103]. Contin et al. [93,104] highlighted the progressive reduction in levodopa half-life with worsening of the disease over the years and with a negative correlation with the severity of symptoms. The authors proposed the computed half-life of levodopa as an indicator of nigrostriatal dopaminergic functionality and integrity [93,104]. In the work of Triggs and Charles [100], a higher degree of drug receptor occupancy and receptor desensitization indicated an advanced disease stage, while Adamiak and Kaldonska [94] demonstrated a correlation between disease duration and EC50. It has been further postulated that higher EC50 values are underpinned by most advanced disease stages, associated with dyskinesias and motor fluctuations [100,105]. Finally, as highlighted by Adamiak and Kaldonska [94], EC50 itself suggests drug sensitivity, while its changes can express dopamine availability. 

### 2.7. Neurophysiology

Neurophysiology techniques are useful to understand the pathophysiology that underlies LIDs. Non-invasive techniques, like electroencephalogram [106,107,108], let one explore the cortical oscillations related to Parkinson’s disease, while invasive techniques, like local field potential (LFP) recordings through deep brain stimulation (DBS) electrodes, provide a more informative insight into basal ganglia’s pathological oscillations [11].

**Figure 4:**
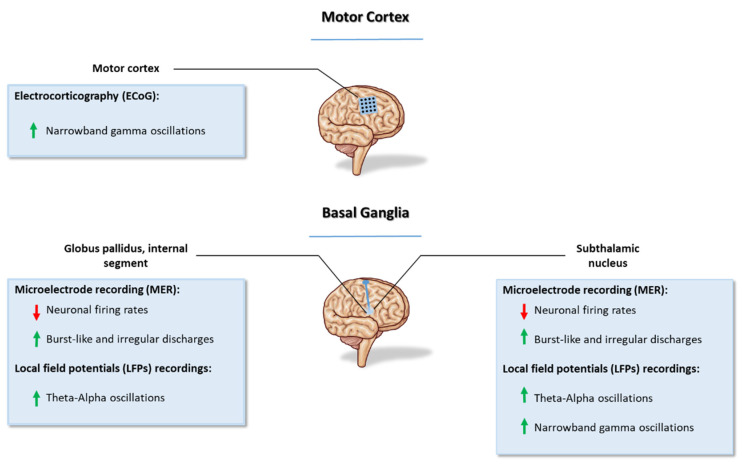
LID neurophysiology findings at cortical and basal ganglia level (for whole description and references, see the corresponding paragraph: 2.7. Neurophysiology).

Indeed, LIDs have been associated with changes in the electrophysiological activity of the motor cortex and basal ganglia circuitry. Both in vivo single-cell recordings and LFPs highlighted the main characteristics of the neuronal firing pattern during LIDs. Single-cell recording using microelectrodes provides information about the frequency and pattern of the discharge of single neurons [109]. The classical model of the basal ganglia function considers LIDs as a result of the over-decreased neuronal firing rates of the globus pallidus internus (GPi), leading to the increased activity of thalamocortical motor circuits [110,111]. These observations have been confirmed in human studies in intra-operatively induced dyskinesias by the administration of apomorphine during pallidotomy in PD patients. A reduction in GPi firing rate was recorded with respect to the OFF state [112,113,114], while no difference was observed between the ON and dyskinetic states [112,113]. The difference in neuronal firing activity concerns not only the frequency but also the pattern of discharge. In ON-state dyskinesias, an increment of burst-like and irregular discharges was observed compared to the OFF state [112,113,114]. Regarding subthalamic nucleus (STN) recording, the mean firing rate of the neurons was not reduced during the ON state without dyskinesias compared to the OFF state. Conversely, it was significantly reduced during LIDs with an increment of spikes in a burst that was absent in the ON state without dyskinesias [113]. The role of the globus pallidus externus (GPe) in LIDs is still unclear. Lozano et al. [115] showed an increment in the firing rate of 50–90%, but further confirmations are needed [115]. The in vivo recording of neuronal oscillatory activity in the GPi, STN and substantia nigra pars reticulata (SNr) is obtained through implanted DBS electrodes. In the STN, peak-dose dyskinesias were associated with an increment in the power of the theta-alpha (4–10 Hz) band with a mean frequency of 8.38 Hz [16,116]. This finding is quite specific and has been confirmed by the increment of the power in the theta-alpha band only when patients exhibited dyskinesia and not during the ON period without dyskinesia. Moreover, in patients with unilateral dyskinesias, this kind of oscillatory activity has been recorded only in the contralateral STN [16]. Patients with diphasic dyskinesias present the same type of oscillatory activity [17]. Peak-dose dyskinesia was associated with theta-alpha activity recorded through electrodes in the dorsal portion of the STN, also known as the motor region [117]. Concerning the GPi, a negative correlation was observed between LFP power in the 8–40 Hz band and the beta bands and LIDs in two patients [118]. LFP oscillations within and coherence between the GPi and STN at low frequencies (<10 Hz) are observed only contralateral to the side of dyskinesia [116]. On the contrary, beta oscillatory activity correlated with the parkinsonian state, rigidity and bradykinesia [119,120]. Recordings of the SNr showed a pathological plasticity and a loss of ability to depotentiate at the output nuclei in patients with dyskinesias [121]. The relationship between hypersynchronization in the gamma frequency band and levodopa-induced dyskinesias has been recently unveiled in the literature [12,13,14,15,16,17,18,19], and the attention on this topic has increased significantly due to its possible clinical and therapeutic implications. For example, Swann and de Hemptinne [12] explored the neuronal activity patterns of dyskinesia by applying a totally implanted multisite brain-recording device in two PD patients treated with DBS, followed over one year. Motor cortex electrocorticography (ECoG) and STN LFP recordings showed that the dyskinetic state was associated with the emergence of a narrowband gamma oscillation both in the motor cortex and STN, pointing out a strong phase coherence. Furthermore, in the study of Halje and Tamte [13], cortical and striatal signals were recorded in a hemiparkinsonian 6-OHDA-induced rat model [122]. Abnormal involuntary movements were neurophysiologically underpinned by a resonant LFP oscillation at 80 Hz in the motor cortex and the striatum of the lesioned hemisphere 10–20 min after receiving intraperitoneal levodopa formulations. Interestingly, this narrowband oscillation was not detectable in the non-dyskinetic animals either ON or OFF levodopa or in the intact hemisphere of any animal [13]. In the same toxin-induced mouse model, Güttler and Altschüler [14] investigated the association of M1 ECoG and motor performance in rats during 21 days of daily treatment with levodopa/benserazide. After levodopa administration, subsequent involuntary movements were accompanied by an increase in cortical narrowband high-gamma oscillations above M1 with an average frequency of 97 Hz. The authors further showed that the gamma power spectrum significantly correlated with the clinical score for abnormal involuntary movements [14]. Narrowband gamma oscillations are not peculiar to levodopa-induced dyskinesias in PD, since such oscillatory activity has been proven also in patients affected by dystonia and myoclonus epilepsy at rest [14,123,124]. A brief overview on the neurophysiological markers of LIDs is schematically depicted in Figure 4.

### 2.8. Neurotransmitter Systems

Insights for the understanding of LID pathophysiology should arise from recent evidence on neurotransmitter modulatory systems that may influence the dopaminergic transmission under peculiar circumstances. Non-dopaminergic pathways can regulate the dopaminergic transmission of the basal ganglia with direct and indirect mechanisms or can be additionally implied in the metabolism of dopamine after the degeneration of dopaminergic neurons. This topic is particularly demanding to explore, since serotonergic, glutamatergic, noradrenergic, cholinergic, opioid, endocannabinoid and adenosinergic systems have a variable, controversial and only partially explained relationship with LIDs. Recent trends agree on the possible role played by such pathways in LID occurrence and maintenance [47], but a complete understanding of the topic is far from being reached, and further investigations are still needed. The following paragraphs summarize a brief overview on how and when these neurotransmitter pathways interplay in LID pathogenesis. Table 3 depicts an overview on neurotransmitter systems.

#### 2.8.1. Serotonergic System

The serotonergic system originates from the raphe nuclei and, through its cortico-subcortical projections, modulates cognition, vegetative functions and movement [125,126]. As described by Lavoie and Parent [127] and Fox and Chuang [128], the influence on motor control should be attributed to the dense serotonergic innervation of the striatum, SNr and GP. Indeed, serotonergic neurons show biochemical similarities with the dopaminergic ones: they share the vesicular monoamine transporter 2 (VMAT2) and AADC enzyme, which catalyzes the decarboxylation of aromatic amino acids. Through this enzymatic machinery, these neurons can convert also exogenous levodopa into dopamine and subsequently release it in an activity-dependent fashion [129,130,131]. Such possibility to generate dopamine with non-dopaminergic terminals is thought to be the cornerstone of serotonergic influence on LIDs. Additionally, movement regulation and LID induction seem exploited by the serotonergic system through the 5-HT1A, 5-HT1B, 5-HT2A, 5-HT2C and the 5-HT3 receptors. Further evidence proved that removing the forebrain serotonergic innervation by the selective toxin 5,7-dihydroxy-tryptamine (5,7-DHT) almost completely suppressed abnormal involuntary movements [132,133,134]. Finally, it has been extensively demonstrated that increasing the serotonergic tone, by the administration of either selective serotonin reuptake blockers (SSRIs) or the serotonergic precursor 5-hydroxytryptophan, significantly reduced LIDs in hemiparkinsonian rats, without compromising the levodopa therapeutic efficacy [135,136,137]. Keeping in mind that serotonergic and dopaminergic neurons are biochemically related, the contribution of the serotonergic side is beneficial when sufficient dopaminergic terminals are spared, since dopaminergic terminals provide a buffering system for the levodopa-derived dopamine. Conversely, as the disease progresses, the contribution of serotonergic neurons becomes detrimental because serotonergic neurons lack an autoregulatory feedback mechanism for dopamine release. As a consequence, levodopa-derived dopamine is released in an uncontrolled way following levodopa administration. 

#### 2.8.2. Glutamatergic System

Glutamate is the major excitatory neurotransmitter in the nervous system, and its transmission depends on three receptor subtypes globally expressed in cortico-subcortical structures: metabotropic receptors coupled to second messenger systems through G-proteins (mGluR), Ionotropic Glutamate N -Methyl-D-Aspartate (NMDA) and Ionotropic Alpha-Amino-3-hydroxy-5-methyl-4-Isoxazolepropionic Acid (AMPA) receptors. As PD progresses, dopaminergic sprouting and reduced DA uptake preserve intrastriatal DA levels [138] with a consequent detrimental glutamatergic control [139,140,141,142]. Dopaminergic decrease and uncontrolled replacement with levodopa alter the glutamatergic transmission within the basal ganglia. Accordingly, corticostriatal glutamatergic activity has been demonstrated to be dramatically increased in PD mouse models [143,144,145]. However, interactions between dopaminergic and glutamatergic systems are complex, since glutamate receptors have a pivotal role in synaptic plasticity, and both postsynaptic changes and the trafficking of the glutamate metabotropic and ionotropic receptors in the synaptic cleft should influence the pathogenesis of LIDs [146]. The three glutamate receptor subtypes are modulated differently throughout the course of PD, and their possible role in LIDs is difficult to establish. For example, group I mGluRs antagonists reduced DA-dependent striatal synaptic plasticity, in terms of both long-term depression (LTD) and long-term potentiation (LTP), raising the possibility of detrimental effects on striatal-dependent motor and cognitive activity [147,148,149]. PD progression additionally influences corticostriatal NMDA-mediated glutamatergic signals, because corticostriatal plasticity depends on either the nigral denervation or differential composition of striatal NMDA receptor subunits [150,151,152]. Differently from mGluR and NMDA receptors, less is clear about the dysregulation of AMPA receptors in PD, and its possible causative role in LIDs is still debatable [142,153,154,155,156]. To conclude, the glutamatergic system is complex and has a plethora of functions that are compromised in a variable fashion in advanced disease stages and appear to be far from completely understood. The literature is controversial on the topic, but it is well established that the chronic levodopa treatment compromises the dopaminergic control on glutamatergic transmission in the long term.

#### 2.8.3. Noradrenergic System

The noradrenergic innervation of the central nervous system (CNS) depends on two main ascending systems. A major source of noradrenaline (NA) is the locus coeruleus (LC), located along the fourth ventricle in the pons, which provides projections to the SNc [157] and striatum [158]. A secondary source of NA is represented by the medullary noradrenergic system, composed of scattered groupings of noradrenergic neurons in the ventrolateral reticular formation and the nucleus of the solitary tract, with primary vegetative functions [159,160,161]. Despite the results of Alachkar and Brotchie [162] and Ribas and Miralles [163], how the noradrenergic system varies throughout the course of PD is a question still open. The NA-synthesizing enzyme DA-β-hydroxylase (DBH) is crucial for NA synthesis and has been exploited for laboratory models: DBH knock-out mice do not produce NA and exhibit both parkinsonism and spontaneous dyskinesias, even if striatal DA is preserved [164]. However, NA loss in PD mouse models has been poorly evaluated in the literature, since in the conventional PD 6-OHDA-induced rat model, the NA transporter (NAT) is blocked prior to 6-OHDA infusion to prevent noradrenergic cell loss. For this reason, only a few studies investigated the effect of additional noradrenergic lesions on LIDs, providing controversial effects on LID severity and duration [165]. For example, direct LC infusions of ibotenic acid reduced LIDs in 6-OHDA-lesioned rats that had previously been rendered dyskinetic [166]. Interestingly, Buck and Ferger [167] demonstrated that exogenous NA, infused in the striatum of hemiparkinsonian rats, elicited LIDs, while Arai and Tomiyama [168] investigated the activity of NAT for DA re-uptake and hypothesized a possible inhibitory role in LIDs. From a receptorial point of view, it is noteworthy that agonists and antagonists of α and β receptors gained attention in recent years due to the possibility to modulate dopaminergic transmission, with variable results. For example, Sommermeyer and Frielingsdorf [169] reduced LIDs with an α-1 receptor antagonist in rodents. Additionally, α-2 receptor antagonists prevented severe/disabling LIDs [170,171,172,173]. Finally, Carpentier and Bonnet [174] demonstrated a reduction in LIDs in humans with propanolol, a β-1/2 receptor antagonist, while β-2 receptor selective antagonists have never been tried for LID management.

#### 2.8.4. Cholinergic System

Nicotine interacts with the nAChRs, ligand-gated ion channels, whose endogenous neurotransmitter is acetylcholine. The most represented nAChR subtypes in regions such as the cortex, hippocampus, thalamus and cerebellum are the α4β2* and α7* nAChRs, while the primary ones in the basal ganglia are the α4β2* and α6β2* subtypes with α7* nAChRs less densely expressed [175,176,177]. An overwhelming body of evidence concluded that nicotine has an established anti-dyskinetic effect, probably mediated by nAChR desensitization/downregulation, with a secondary reduction in striatal dopamine release. In fact, nicotine administration has been proven to alleviate both peak and total LIDs by approximately 60% in MPTP-lesioned nonhuman primates (NHPs) for 30 weeks [178,179,180], and its readministration, after a 10-week washout period, led to an immediate decline in LIDs. Additionally, mice lacking both α4β2* and α6β2* nAChRs had reduced baseline LIDs, suggesting a role as an LID primary regulator for the β2 subtype [181,182,183]. However, it is likely that multiple nAChR populations influence LIDs, including the α4β2* α6β2* and α7 subtypes. Since multiple compensatory changes occur throughout the course of PD, various nAChR subtypes may be differentially related to LIDs during disease progression. The cholinergic system also includes muscarinic receptors mAChRs, numbered from M1 to M5 and coupled to G-proteins [184,185]. These receptors are highly expressed in the striatum [184,185] and do not have an established role in abnormal involuntary movements: atropine had no effect on LIDs [186], while the muscarinic antagonist dicyclomine reduced LIDs in 6-OHDA-lesioned mice.

#### 2.8.5. Opioid System

Endogenous opioid peptides, except endomorphins, share the amino acid enkephalin sequence at the N-terminus, with differing extensions at the C-terminus [187]. Opioid receptors are widely distributed across cortical and subcortical structures of the CNS, such as the basal ganglia, nucleus accumbens and ventral tegmental area. Opioid receptor distribution and activity undergo variable modifications throughout the course of PD [187]. μ-receptor levels are reduced in the striatum and GPi, κ-receptor levels are reduced in the GPe and GPi, while δ-receptor levels are unchanged in the striatum of dyskinetic NHPs. Moreover, signaling is overactive for μ, δ and κ-receptors, respectively, in the striatum, GPi and caudate nucleus and motor cortex of MPTP-lesioned NHPs. Two seminal studies investigated opioid levels in dyskinetic models and found elevated levels of dynorphin B and α-neoendorphin in the dorsolateral striatum [188] and SN [189] of severely dyskinetic rats compared with mildly dyskinetic or non-dyskinetic rats, while dynorphin A has been found elevated in dyskinetic nonhuman primates by Bourdenx and Nilsson [190]. Non-selective opioid receptor antagonists generated ambivalent results for LID control [191,192,193]. Such a lack of clear anti-dyskinetic actions is thought to reflect the interaction of non-subtype-selective ligands with multiple opioid receptors, providing competing pro- and anti-dyskinetic effects. The leading theory in the field considers the blockage of μ and δ receptors as anti-dyskinetic, while the blockade of κ-opioid receptors should promote LIDs [194].

#### 2.8.6. Endocannabinoid System

The endocannabinoid system consists of a family of lipid signaling molecules released on demand from membrane lipid precursors and the relative biochemical enzyme machinery involved in their synthesis and degradation [195,196]. Arachidonoyl ethanolamine (anandamide) [197,198] and 2-arachidonoyl glycerol (2-AG) [199] represent the progenitors of this group of molecules, but the number of new members is rapidly increasing [200]. The main receptors are coupled to G-proteins (CB1 and CB2), while others belong to the transient receptor potential (TRP) family, as well as nuclear peroxisome proliferator-activated receptors (PPAR) [201]. CB1 receptors are represented on GABAergic striatofugal neurons [202,203] and the subthalamic nucleus [204]. The endocannabinoid system has pleiotropic functions in movement: an increased endocannabinoid transmission reduces striatal glutamate release, relieving PD symptoms [143,205], while the activation of CB1 on striatofugal terminals may empower the indirect pathway, amplifying the inhibitory output of the basal ganglia. However, it is a well-known fact that these molecules counterbalance the dopamine-mediated hyperactivity [206,207,208]. LID improvement is attributed to the reduction in the levodopa-induced sensitization of dopamine receptors, normalization of aberrant glutamate release and rebalancing of the maladaptive plasticity in the denervated striatum. In support of this hypothesis, several attempts have shown cannabinoid-mediated improvement in levodopa-induced abnormal involuntary movements in rodent models and NHPs [206,209,210,211,212] and PD patients [213], avoiding global motor suppression [206]. CB1 receptors are also expressed on serotonergic raphe-striatal fibers [214], through which they can act as pseudodopaminergic neurons for abnormal dopamine storage and release, contributing to LID development, as mentioned before (see Section 2.8.1) [132]. To conclude, it is likely that the endocannabinoid system may promote anti-dyskinetic effects, by both dampening the ectopic dopamine release from serotonergic terminals and inhibiting 5-HT release [215,216].

#### 2.8.7. Adenosinergic System

Adenosine, a ubiquitous endogenous nucleoside, is a modulator of neurotransmission exerted by DA, glutamate and acetylcholine and has been implicated in reward-related behavior [217,218,219,220,221,222,223]. Adenosine receptors are widely expressed in the CNS: A1 subtypes are distributed in the cortex, hippocampus and cerebellum, A2A are mainly distributed in the striatum and olfactory bulb, while A2B and A3 subtypes are found at low levels of expression. The activation of adenosine A2A receptors in the striatum regulates dopamine and glutamate release. Adenosine A1 receptors generate functionally interacting complexes in cortical neurons and the basal ganglia [224,225]. A higher number of these receptors has been found in the striatum in dyskinetic patients compared to PD patients without LIDs during autopsy studies. The role of adenosine in LID management has not been primarily investigated so far, and improvement in motor function and OFF time were the primary endpoints in the few studies that tested adenosine A2A receptor antagonists [226,227,228,229,230,231,232,233]. 

However, specific studies addressing LIDs have not been conducted yet, and a direct anti-dyskinetic effect seems unlikely.

### 2.9. Imaging Studies 

**Figure 5:**
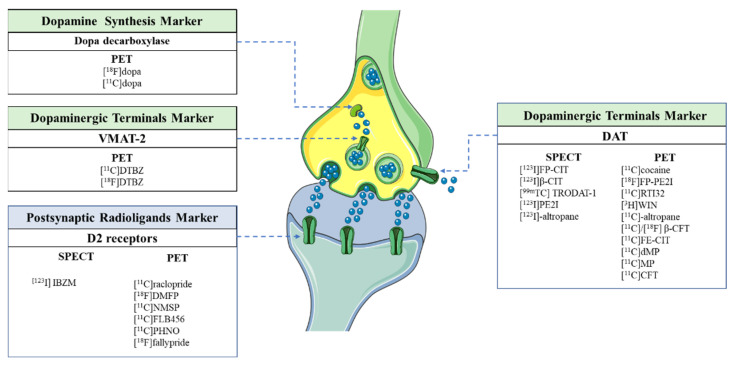
Nuclear neuroimaging ligands for Parkinson’s disease studies. Legend: DAT: Dopamine Transporter; PET: Positron Emission Tomography; SPECT: Single Photon Emission Computed Tomography; VMAT-2: Vesicular Monoamine Transporter 2. (The figure was partly generated using Servier Medical Art, provided by Servier, licensed under a Creative Commons Attribution 3.0 unported license).

Dopaminergic function can be assessed in vivo using neuroimaging studies [234] with specific ligands for dopamine receptors, the VMAT2, the plasmalemmal DAT [235,236] and postsynaptic DA D1R and D2R receptors. Moreover, the uptake and decarboxylation of levodopa to DA, the storage of DA and the DA turnover could be assessed using the fluorinated analog of levodopa, [^18^F]fluoro-L-dopa [237], as shown in Figure 5. 

Clinical observations of the prevalence of dyskinesias in more advanced disease stages found evidence in a report showing an inverse relationship between [^18^F]fluoro-L-dopa uptake and dyskinesias [238]. Although presynaptic DA denervation plays a crucial role in the pathogenesis of dyskinesia [239], it is not the only factor involved in the development of dyskinesia, and the pattern of DA receptor stimulation plays a key role too. Patients who developed motor fluctuations showed a greater magnitude but a less sustained decline in [^11^C]Raclopride binding, with respect to patients with a stable response [240]. Moreover, the relative reduction in [^11^C]Raclopride binding 1 h after oral levodopa intake advances with disease duration and is more pronounced in patients suffering from LIDs compared to those with a stable response. Moreover, no difference was found between dyskinetic and non-dyskinetic subjects 4 h after levodopa, adding evidence of a more pulsatile pattern of levodopa in subjects with motor complications [240,241]. Similar findings have been observed by other groups [242]. Another marker of advanced disease and a risk factor for dyskinesia onset is the increase in DA turnover. Prolonged scans with [^18^F]D measure uptake at 90–120 min and reflection uptake, and decarboxylation to fluoroDA and the trapping of fluoroDA in synaptic vesicles also reflect the egress and subsequent metabolism of this trapped radioactivity. The effective distribution volume, which is derived from this reversible tracer model, correlates well with the inverse of the ratio of tracer loss to tracer uptake constants [237], which in turn correlates with classical neurochemical measures of DA turnover [243]. DA turnover measured using this approach is increased early in PD [244], and further increases occur with disease progression [245]. Sossi and de la Fuente-Fernandez [246] investigated the possible age dependency of changes in DA turnover, in terms of DA distribution volume, as a contributing factor to the levodopa-related complications, with the analysis of the plasma input uptake rate constant (Ki) after [^18^F]D uptake, an indicator of DA synthesis and vesicular storage capacity. The authors concluded that the magnitude of the increase in DA turnover was greater than the magnitude of the decrease in DA synthesis and storage rate in PD patients with younger disease onset, in line with an increased susceptibility of these patients to develop motor complications [247,248,249,250]. DAT downregulation plays a role in the DA turnover; however, serotonergic neurons are able to convert exogenous levodopa into DA too, with a non-regulated release and uptake of DA. The DA system is not the only actor implied in dyskinesia development, since other neurotransmitters play a role too. Piccini and Weeks [251] demonstrated reduced striatal binding of the opioid ligand [^11^C]Diprenorphine in PD patients with LIDs, presumably reflecting the occupancy of striatal opioid receptors due to increased opioid levels. Studies have demonstrated increased adenosine A2 binding in PD patients with abnormal involuntary movements compared to those dyskinesia-free [252]. Even brain hemodynamics seem to be influenced by levodopa. Hirano and Asanuma [253] used a multimodal PET to image both blood flow (rCBF) and glucose metabolism in the same scanning session: a dissociation between flow and metabolism was found in patients affected by LIDs after the administrations of oral levodopa. Interestingly, during the OFF-medication state, regional glucose metabolism and rCBF matched; conversely, after the administration of oral levodopa, rCBF was greatly enhanced while glucose metabolism was unchanged in the network composed of putamen, pallidum and midbrain pons. Flow/metabolism dissociation was greater in LID patients, supporting the hypothesis of a hemodynamic effect of levodopa. The underlying mechanisms of such evidence have not been solved yet. An increased capillary density in the striatum and midbrain that may drive more blood flow to these regions under the conditions of a high DAergic tone [254,255] is the leading hypothesis in the field so far. Finally, it is worth mentioning the serotonergic contribution to LIDs, since it gained increasing attention in the last years. As mentioned in the “Serotonergic system” paragraph, putaminal serotonergic fibers have the potential to store and release dopamine in a non-physiological manner, promoting LIDs, and this contribution has been further investigated with radioligand tracers. For this purpose, Lee and Seo [256] enrolled 30 patients with PD, classified as dyskinetic, non-dyskinetic and drug-naive and acquired two PET scans and 3T MRI scans for each patient using [(11)C]-3-amino-4-(2-dimethylaminomethylphenylsulfanyl)-benzonitrile (^11^C-DASB), a ligand of the serotonin transporter, and N-(3-[(18)F]fluoropropyl)-2-carbomethoxy-3-(4-iodophenyl) nortropane (^18^F-FP-CIT), a DAT radioligand. The ^11^C-DASB/^18^F-FP-CIT ratio was computed to estimate serotoninergic fiber innervation relative to dopaminergic fiber availability. The study showed the highest ^11^C-DASB/^18^F-FP-CIT ratio in the putamen and pallidum for dyskinetic PD patients, highlighting the pivotal role of the serotonergic innervation in LIDs.

## 3. Therapeutic Options

Different therapeutic approaches have been proposed in order to face the LID problem [257,258,259]. On the pharmacological side, one possible solution is to avoid the pulsatile administration of levodopa, through a continuous administration of levodopa [260,261,262,263,264] or dopamine agonists like apomorphine [265]. Other pharmacological options include levodopa regimen optimizations in terms of doses and inter-dose timing and the usage of long-release drugs or add-on medications. The aim is to flatten the levodopa fluctuations. However, neurons are responsive to pharmacological stimulation but also to electrical [266], magnetic [267] or ultrasound stimulations [268,269]. Among stimulation techniques, invasive DBS has been used for the treatment of both hyperkinetic [270,271,272,273] and hypokinetic movement disorders, like Parkinson’s disease [274,275,276,277]. 

### 3.1. Levodopa Therapy Optimization

**Figure 6:**
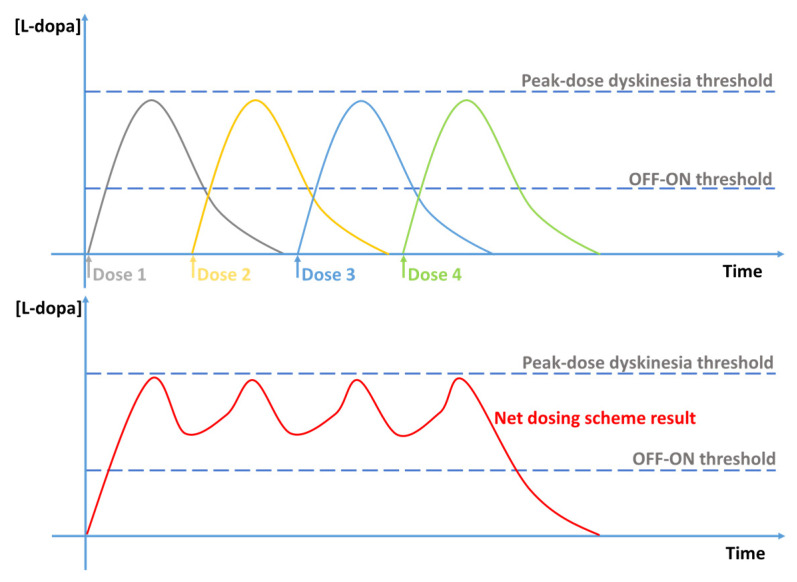
Upper graph: pharmacokinetic of 4 single doses of levodopa without the computation of the previous doses (Doses 1 to 4). Lower graph: real pharmacokinetic of the same 4 single doses of levodopa describing the net effect of the therapeutic scheme (the net dosing scheme is the result of the combination of Doses 1 to 4). Legend: lower dashed line = levodopa concentration threshold for OFF–ON transition; upper dashed line = levodopa concentration threshold for peak-dose dyskinesia transition.

The aim of the therapies for LIDs is to reduce the fluctuations of levodopa levels and improve the pharmacokinetics of levodopa by the prevention of dopamine catabolism and the usage of controlled-release levodopa or dopamine agonists [109]. It has been hypothesized for a long time that providing a more continuous and constant levodopa administration could reduce the risk of motor complications, even in early PD patients. This idea was tested in the STRIDE-PD trial, conducted by Stocchi and Rascol [28]: the risk of developing dyskinesias was compared during a 134-week double-blind trial in 747 PD patients randomized to levodopa/carbidopa (LC) or levodopa/carbidopa/entacapone (LCE). It is worth noting that early PD patients on LCE failed to delay the onset of motor complications: the LCE group was associated with a shorter time to onset and an increased frequency of LIDs. For the selection of the correct therapeutic scheme, the pharmacokinetic/pharmacodynamic of each dose of levodopa should be kept in mind to guide the selection (Figure 6).

It is worth noting that the therapeutic approach depends on the type of dyskinesia itself.

For peak-dose and square-wave dyskinesias, the following therapeutic approaches can be considered [47,278,279,280]:

- Reducing the dose of levodopa and distributing the inter-dose timing.
- Adding an add-on medication, such as Amantadine, can help to reduce the severity of dyskinesias.
- For diphasic and square-wave dyskinesias, the following therapeutic approaches can be considered [47,278,279,280]:
- Adjusting the timing of medication doses: spreading out the doses throughout the day can help to maintain more stable medication levels.
- Adding an add-on medication, such as Amantadine, can help reduce the severity of dyskinesias.

For OFF dystonia, the following therapeutic approaches can be considered [47,278,279,280]:

- Adjusting the timing and dosage of medication or increasing the dose of levodopa can help maintain more stable medication levels.
- Adding an add-on medication, such as an extended-release dopamine agonist, MAO-B inhibitor or COMT inhibitor, can help to reduce the severity of OFF dystonia by potentiating the dopaminergic stimulation.
- Apomorphine injections or sublingual administration [281] can provide rapid relief from OFF dystonia.

All types of LIDs can benefit from continuous levodopa or Apomorphine administration or deep brain stimulation. 

A successful approach to improve pharmacokinetics includes alternative routes of drug delivery to bypass the delayed gastric emptying [282,283]. More constant plasma levodopa concentrations are achieved through a gelified version for the intrajejunal administration of levodopa/carbidopa, in patients with advanced PD [284,285]. The efficacy of this formulation has been confirmed by a randomized controlled trial [260]. It is worth noting that the GLORIA registry, a 24-month, non-interventional and observational registry, conducted by Antonini and Poewe [286], was the study with the largest cohort of PD patients treated with levodopa/carbidopa intestinal gel (LCIG). In particular, in this registry, the authors investigated the impact of such device-aided therapy on advanced PD. Results demonstrated improvements in motor fluctuations and non-motor symptoms, such as sleep, mood and the quality of life. The more frequent adverse effects were weight loss (6.7%), device-related infections (5.9%), device dislocations (4.8%) and polyneuropathy (4.5%).

The evolution of LIDs on LCIG therapy deserves a separate discussion. Unexpectedly, a therapy with LCIG could not completely abort LIDs, but dyskinesias may vary and change their profile throughout the course of the disease, despite an appropriate therapeutic regimen. While a reduction in motor fluctuations is predictable, less is known about how the LID profile is modulated by such therapy. For this purpose, Szász and Constantin [287] investigated dyskinesia features in advanced PD patients before and after 6, 12 and 18 months from LCIG. As expected, motor fluctuations improved, but abnormal involuntary movements changed their pattern. In fact, 18 months after LCIG positioning, severe peak-dose dyskinesias dropped from a mean of 1.61 o 0.04 h, while the mild/moderate ones increased from a mean of 1.97 to 2.79 h. Diphasic dyskinesias were reduced from an average of 4.03 to 1.81 h, and dystonia dropped from a total of 12 to 9 h per day. This work highlights the need for advanced PD patients on LCIG to receive a customized and targeted therapeutic approach [287].

Other approaches to improve pharmacokinetic parameters include the use of extended-release formulations of levodopa [288,289,290], the administration of levodopa gastric retention formulation [282] and the combination of immediate- and extended-release formulations with gastric retention [283]. 

### 3.2. Non-Dopaminergic Drugs

Non-levodopa-based therapeutic strategies for dyskinesias rely on the possible role of non-dopaminergic systems, such as the serotonergic ones, for the induction of LIDs [291,292]. The 5-HT1A agonist Buspirone was effective in reducing LIDs after oral administration and reduced levodopa-evoked striatal synaptic dopamine release [291,292]. Another Serotonin 5-HT1A agonist used to manage LIDs is Sarizotan, whose potential feasibility for the scope was not confirmed in a large randomized, placebo-controlled, phase IIb trial [293]. 5-HT1A/1B receptor agonists Eltoprazine and Anpirtoline [294,295] revealed efficacy in reducing LIDs in animal models, while the results were not superior to Amantadine in a translational human study [296]. NMDA antagonists, like Memantine, Remacemide, Dextromethorphan, Milacemide and CP-101.606, were assessed as therapeutic options, without clinical evidence of any anti-dyskinetic effect in experimental models of LIDs [297,298,299,300]. Antiepileptic drugs were tested too with poor results. For example, Gabapentin showed no difference with respect to the placebo in reducing dyskinesias [301]. Moreover, a randomized double-blind, placebo-controlled, parallel-group trial with Levetiracetam failed to show a significant reduction in dyskinesias [302]. Zonisamide at the dosage of 50 mg decreased dyskinesias, but dizziness, apathy and weight loss were side effects reported by the patients [303]. Among antipsychotics, Clozapine, Olanzapine and Quetiapine were the most studied. A large, randomized, placebo-controlled trial with Clozapine showed a reduction in dyskinesias with increased ON time without dyskinesias and no effect on increasing OFF time or increased adverse events [304]. Quetiapine failed to show benefits in dyskinesias compared to placebo [305]. Finally, Olanzapine revealed some anti-dyskinetic effects during a randomized, placebo-controlled, cross-over trial but also increased OFF time [306]. 

### 3.3. Deep Brain Stimulation

Another possible therapeutic option is DBS, targeting either the GPi or STN. When the target is the GPi, DBS has a direct effect on LID reduction; conversely, when the STN is chosen, involuntary movements are reduced through an indirect effect on dyskinesias by lowering levodopa thanks to the antiparkinsonian effect of STN stimulation. DBS can lead to new possibilities for LID management, due to the high informative value of basal ganglia oscillatory activity about the motor state and its potential reliability to be real-time adjusted for symptom control [86]. 

### 3.4. Closed-Loop Therapy

**Figure 7:**
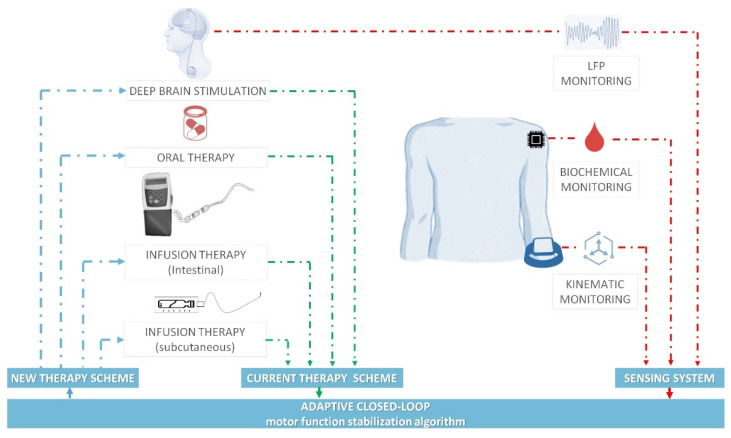
Adaptive closed-loop therapy system for Parkinson’s disease patients. The four therapies, alone or in combination (deep brain stimulation, oral therapy, intestinal infusion therapy and subcutaneous infusion therapy) can be administered in adaptive controlled closed-loop way, through a multiparametric (LFP, biochemical and kinematic monitoring) sensing system. (Reproduced, under the terms of the Creative Commons Attribution 4.0 License, from [86]).

Predictors of motor performances can be used as feedback to real-time fit and optimize therapeutic regimens in a closed-loop fashion to restore a physiologic dopaminergic stimulation pattern and limit dyskinesias in PD patients [86]. An adaptive closed-loop administration algorithm improves dyskinesias by reducing the fluctuations in dopamine levels and reproducing the normal dopaminergic tone. Biochemical, neurophysiological and wearable sensors are sensing systems expected to provide feedback signals to close this loop. A multiparametric modular sensing system that combines biochemical, neurophysiological and wearable sensor data could adapt the administration of different combinations of antiparkinsonian therapies in real time, as shown in Figure 7 [307,308,309]. Biochemical and neurocomputational models of the levodopa pharmacokinetics and dynamics were shown to predict the motor response with a varying levodopa plasma concentration in both stable and fluctuating PD patients [93,310,311,312,313], and a sigmoid curve was demonstrated to express the relationship between levodopa plasmatic concentration and tapping frequency. Biochemical levodopa sensing approaches include detection in blood [314], sweat [313], skin [315], skeletal muscle [314], subcutaneous tissue samples [316] and electrochemical sensing with amperometry or voltammetry [317]. On the neurophysiological side, synchronized oscillatory rhythms reliably associated with hypokinetic and dyskinetic states provide potentialities as a control signal in closed-loop DBS [12,318,319]. Sensing cortical or subcortical brain activity and real-time symptom correlate extraction during DBS is the most advanced closed-loop strategy: basal ganglia beta activity (13–30 Hz) is related to hypokinetic state and bradykinesia and is, to date, the most promising neurophysiological biomarker for closed-loop DBS in PD [320,321,322], while the basal ganglia activity in the gamma range (25–140 Hz) is the electrophysiological signature of the dyskinetic state [12]. Wearable sensors integrated with machine learning algorithms can convert kinematic motor data into quantitative signals, recognizing and predicting changes in the motor state [323]. 

## 4. Discussion

Since its introduction as an antiparkinsonian drug regimen, levodopa has become the leading therapeutic option for motor control in PD [3]. Abnormal involuntary movements are well recognized as a possible complication secondary to levodopa-based therapy, and it is not only movement disorder specialists that have to frequently face this therapy-induced drawback [38]. This review points out the complexity of their neurophysiological correlates and pharmacokinetic and pharmacodynamic models. The literature discussed in depth the temporal relationship between different antiparkinsonian drugs and dyskinesias [7,21,22,23,24,28,39,40,41,42,43,44,45], highlighting the major role played by levodopa over dopamine agonists [22,23,24,25,26,27], COMT inhibitors [28] and MAO inhibitors [29]. Moreover, LIDs are not stereotyped in nature, since they can be different in phenomenology [46,47,48,49]. Such a complexity is reflected in the basal ganglia firing pattern, modified not only in quantity but also in quality [112,113,114]. An exhaustive pathophysiologic explanation of LIDs is an unmet need so far. Imaging studies reinforced the role of dopaminergic transport, progressive presynaptic terminal denervation and the pattern of DA receptor turnover in the advanced disease stages as possible contributing factors but not as the *primum movens* [239,245,246,247,248,249,250]. Classical therapeutic choices to manage LIDs are limited in efficacy due to the poor feasibility to reach a delicate compromise between excessive and poverty of movement: conventional strategies essentially aim to reduce levodopa fluctuations and stabilize its blood levels to restore a more efficacious and constant dopaminergic tone [47,109,278,279,282,283,284,285]. New therapeutic scenarios will arise from wearable, biochemical and neurophysiological sensing systems, expected to provide feedback signals in a closed-loop system to predict the motor response with a varying levodopa plasma concentration [307,308,309]. The aim of these techniques is to optimize the administration of the currently existing therapeutic options in relation to the real-time motor status.

## Figures and Tables

**Table 1:** Genetic risk factor for LIDs.

Gene	Function	Gene Variant	Ref.
COMT	Involved in the metabolism of dopamine	Val158Met	[30]
DRD2	Encodes the D2 subtype of the dopamine receptor	ANKK1 TTCTA haplotype	[32]
BDNF	Involved in neuronal survival and synaptic plasticity	Val66Met	[34]
SLC6A3	Dopamine transporter gene (DAT)	40-bp VNTR	[31]
GRIN2A	Encodes a subunit of the NMDA receptor, involved in glutamate transmission	rs7192557 and rs8057394	[33]

Legend: BDNF, brain-derived neurotrophic factor gene; COMT, catechol-O-methyltransferase; DRD2, dopamine receptor D2 gene; GRIN2A, N-methyl- d -aspartate receptor subunit 2A; SLC6A3, dopamine transporter gene.

**Table 2:** Relationship between the incidence of levodopa-induced dyskinesias and levodopa exposure time.

Type of Study	N° Patients	Levodopa Exposure	% Dyskinetic Patients	Ref.
Meta-analysis	335 (pre-levodopa era)	3–6 w	23.3	[41]
	606 (pre-levodopa era)	2–4 m	34.5	
	606 (pre-levodopa era)	5–6 m	53.4	
	2645 (pre-levodopa era)	7–12 m	54.8	
	982 (pre-levodopa era)	1–2 y	71.9	
	297(pre-levodopa era)	2.5–3.5 y	56.7	
	432	7–12 m	7	
	575	1–2 y	28.7	
	747	2.5–3.5 y	26.9	
	1599	4–6 y	36.2	
	514	9–15+ y	87.8	
Prospective, double-blind, randomized clinical trial	88	5 y	45	[24]
	27	10 y	77.8	
Clinico-pathological	42	6.4 y	31	[39]
		14.3 y	61.9	
Community-based	87	<5 y	11	[7]
		6–9 y	32	
		>10 y	89	
Community-based	126	5 y	30	[40]
		10 y	59	
DATATOP	352	20.5 m	30	[42]
FIRST	187 on IR	5 y	20.6	[43]
	193 on CR	5 y	21.8	
056-study	45	5 y	45	[23]
CALM-PD study	131	3 y	54	[44]
PELMOPET study	90	3 y	26	[22]
ELLDOPA study	92 (150 mg/die)	8 m	3	[21]
	88 (300 mg/die)	8 m	2	
	91 (600 mg/die)	8 m	16	
STRIDE-PD study	372 (Ldopa)	2 y	32	[28]
	373 (Ldopa + entecapone)		38	
SIDNEY study	52	15 y	94% (12% severe dyskinesias)	[45]

Legend: w, weeks; m, months; y, years.

**Table 3:** Summary table of the main characteristics of neurotransmitter systems involved in LIDs.

	Neurotransmitter	Receptor	Therapeutic Target	Effect on LIDs
Serotonergic system	Serotonin	5-HT1A 5-HT1B	5-HT1A and 5-HT1B Receptor Agonists	- Density of serotonergic terminals in the striatum directly correlates with the severity of LIDs - Serotonergic neurons convert exogenous levodopa into dopamine and release it without autoregulatory feedback
		5-HT2A 5-HT2C	5-HT2A Receptors Antagonists	
		5-HT3	- Not established - SERT inhibition	
Glutamatergic system	Glutamate	mGluR	MGluR antagonist	- Altered trafficking - Hyperactive
		NMDA	NMDA receptor antagonist (GluN2A/B subunit)	- Altered trafficking - Alteration of subunit composition - Supersensitivity in the putamen following long-term levodopa
		AMPA	AMPA receptor antagonist	- Altered trafficking - Increased index of rectification (IR) of AMPA current in striatal medium spiny neurons - Increased activity of Ca^2+^ -permeable AMPAR due to hyperphosphorylation of GluR1 subunit
Noradrenergic system	Noradrenaline	α-1/2	α receptor antagonist	- NA loss causes parkinsonism and spontaneous dyskinesias in DBH knock-out mice - NA infusion in the striatum promotes LID in hemiparkinsonian rats - NAT activity should re-uptake DA and reduce LIDs - Controversial evidence
		β-1/2	β receptor antagonist	
Cholinergic system	Acetylcholine	nAChR (α4β2* and α6β2* subtypes)	β2* nAChR agonist	β2 subtype reduces LIDs, but nAChR vary over the course of PD
		mAChR (m1 to m5)	Variable results with muscarinic antagonists	Not established
Opioid system	*Enkephalin*	*δ*	δ—receptor selective antagonist	- Elevated levels of dynorphin B, α-neoendorphin and Dynorphin A in the dorsolateral striatum and SN - μ and δ receptors promote LIDs - κ receptor reduces LIDs
	β-endorphin Endomorphin	*μ*	μ—receptor selective antagonist	
	Dynorphin A Dynorphin B α-neoendorphin β-neoendorphin	*κ*	κ—receptor selective agonist	
Endocannabinoid system	Anandamide 2-AG	CB1/2	CB-receptor agonist	- The stimulation of the CB1 receptors reduces LIDs by: ● Desensitization of DA receptors ● Normalizing aberrant glutamate release - Net anti-dyskinetic effect - CB1 receptors can also promote LIDs by dopamine synthesis in serotonergic raphe-striatal fibers
		TRP	Not established	Not established
		PPAR	Not established	Not established
Adenosinergic system	Adenosine	A2A	- A2A receptor antagonist	- Not clearly established, but the activation of this receptor in the striatum regulates amplification of dopamine and glutamate release - A direct anti-dyskinetic effect seems unlikely
		A2B	Not established	Not established (poorly expressed in CNS)
		A3	Not established	Not established (poorly expressed in CNS)

## Data Availability

Not applicable.
